# Effect of long COVID-19 syndrome on health-related quality of life: a cross-sectional study

**DOI:** 10.3389/fpsyg.2024.1394068

**Published:** 2024-05-30

**Authors:** Arthur Nascimento Rodrigues, Alna Carolina Mendes Paranhos, Livia Caroline Machado da Silva, Stanley Soares Xavier, Camilla Costa Silva, Rosilene da Silva, Lidiane Assunção de Vasconcelos, Ivonete Vieira Pereira Peixoto, Tatiana Menezes Noronha Panzetti, Priscila Rodrigues Tavares, Cíntia de Sousa Reis, Beatriz Freitas Launé, Vera Regina da Cunha Menezes Palácios, Pedro Fernando da Costa Vasconcelos, Juarez Antônio Simões Quaresma, Luiz Fábio Magno Falcão

**Affiliations:** ^1^State University of Pará, Belém, Brazil; ^2^Programa de Pós-Graduação em Biologia de Agentes Infecciosos e Parasitários, Federal University of Pará, Belém, Brazil; ^3^School of Medicine, University of São Paulo, São Paulo, Brazil; ^4^Center for Biological Health Sciences, State University of Pará, Belém, Brazil

**Keywords:** post COVID-19 condition, quality of life, headache, sleep quality, anxiety

## Abstract

**Purpose:**

This study aimed to assess the association of anxiety, headache, and insomnia on the QoL of patients with long COVID-19.

**Methods:**

We conducted a cross-sectional survey between August 2020 and March 2023. A total of 200 participants were eligible, 53 were excluded and 147 patients with long COVID were included. QoL was evaluated across eight domains using the 36-Item Short Form Health Survey (SF-36). Standardized protocols including the Beck Anxiety Inventory (BAI) (*n* = 103), Pittsburgh Sleep Quality Index (PSQI) (*n* = 73), and Migraine Disability Assessment (MIDAS) (*n* = 67) were also used.

**Results:**

Participants with sleep disorders had significantly lower Vitality (*p* < 0.001). Participants with anxiety disorders had significantly lower Vitality (*p* = 0.001), poorer Mental Health (*p* = 0.008), and more severe Bodily Pain (*p* = 0.008). Participants with headache had significantly lower Vitality (*p* = 0.032), poorer Mental Health (*p* = 0.036), and poorer Physical Functioning (*p* = 0.016). Participants with both headache and anxiety had significantly lower Vitality (*p* = 0.005) and Mental Health (*p* = 0.043) domain scores. Correlation analysis revealed that higher scores for anxiety, sleep disorder, and headache were independently correlated with poorer QoL across various domains. The presence of sleep disorder was associated with a fourfold increase in risk of experiencing diminished Vitality (odds ratio [OR]4.47; 95% CI 1.01–19.69; *p* = 0.048).

**Conclusion:**

Participants with anxiety, sleep, and headache disorders tended to have a worse QoL. The Vitality and Mental Health domains were the most adversely affected in patients with long COVID. Sleep disorders were associated with a fourfold increase in the risk of poor Vitality.

## 1 Introduction

The long-term effects of COVID-19 are still observed. Long COVID generally has a negative effect on the quality of life (QoL) of COVID-19 survivors (Malik et al., 2022; Román-Montes et al., 2023; WHO, 2023). QoL can be affected by health conditions associated with chronic morbidity (Kuyken, 1995). Some survivors of SARS-CoV-2 infection have several long-term persistent symptoms such as fatigue, anosmia, headache, anxiety, and insomnia (Mendelson et al., 2020; Al-Ramadan et al., 2021; Martelletti, 2021). Individuals with long COVID and headache tend to have severe symptoms, which may limit their daily activities, work, social and family life, and studies (Cavallini et al., 1995; Rodrigues et al., 2023).

Besides the headache, individuals with insomnia may also have reduced ability to perform activities of daily living, including reduced performance at work (Scarpelli et al., 2023). Anxiety disorders are common in patients with long COVID and patients with this condition generally have worse QoL (Rubio et al., 2013, 2014). A study from Egypt showed that anxiety disorders are associated with worse physical and mental components of QoL in patients with long COVID (Abdelghani et al., 2022). Taboada et al. (2021) reported that at least 50% of patients who had recovered from COVID-19 reported functional limitations in their daily life 6 months after hospitalization.

Multiple potential causes may account for the long-term persistence of these neurological symptoms in the context of COVID-19, including sustained dysregulation of neurotransmitters that can result in hyperexcitation, leading to sleep disturbances and anxiety disorders (Riemann et al., 2010; Blake et al., 2018). Nevertheless, there are unique aspects of certain populations that may exacerbate these conditions. In the Amazonian context, the convergence of local factors such as deforestation, fires, climate change, housing conditions, and inadequate basic sanitation among Amerindian populations can exacerbate the severity of long COVID within this community (Santos et al., 2010; Garnelo, 2019; Alves de Oliveira et al., 2021; Codeço et al., 2021; Feng et al., 2021) and adversely impact their quality of life.

The persistence of symptoms in long COVID-19 patients can significantly diminish their QoL due to several factors. Notably, incapacitating headaches, sleep disorder, and anxiety emerge as prominent contributors, with each potentially serving as both cause and consequence of the others. This promotes a vicious cycle in which one affliction may exacerbates another. Therefore, this study aimed to evaluate whether there is a difference in the QoL of patients with the presence or absence of headache, sleep disorders, anxiety in patients with long-term COVID residing in the Eastern Amazon, as well as to identify predictors of worse QoL in these patients.

## 2 Methods

### 2.1 Ethical aspects and study design

This cross-sectional observational study was reported in accordance with the Strengthening the Reporting of Observational Studies in Epidemiology (STROBE) guidelines. It was approved by the Ethics Committee for Human Subjects Research of the State University of Pará (approval number 3,619,141) and was conducted in accordance with the Declaration of Helsinki. All participants provided written informed consent to participate in the study.

### 2.2 Study participants

All patients aged 18 years or older with a diagnosis of long COVID were invited to participate. We followed the diagnostic criteria for long COVID established by Raveendran (2021) which includes a previous diagnosis of symptomatic or asymptomatic COVID-19 with persistence of at least 2 weeks of symptoms and which presented positive polymerase chain reaction tests for SARS-CoV-2 in the acute phase (such as polymerase chain reaction test, serology, or chest computed tomography) and was characterized by fatigue, shortness of breath, cough, joint pain, chest pain, muscle pain, headache or other symptoms that cannot be attributed to another cause. Clinical assessment was performed after symptoms persisted for at least 4 weeks after the acute phase, were present for at least 3 months, with onset of symptoms appearing within 2 months of infection.

The exclusion criteria were: patients with comorbidities that interfere with QoL or its assessment prior to COVID-19 onset, including as chronic obstructive pulmonary disease, heart failure, cardiopathies with hemodynamic repercussions, hypothyroidism, osteoarticular diseases and who did not complete any of the standardized protocols sent by an instant messaging application.

Initially, a face-to-face interview using standardized protocols was carried out in the Post-COVID-19 Program outpatient clinic at the University of Pará State, Belém, Pará, Brazil to collect sociodemographic data, QoL information, and data on symptoms in long COVID-19. Standardized self-completed questionnaires were subsequently sent via instant messaging application to assess QoL (36-Item Short Form Health Survey – SF-36), anxiety (Beck Anxiety Inventory - BAI), insomnia (Psychometric Analysis of the Pittsburgh Insomnia Rating Scale - PSQI), and headache (Migraine Disability Assessment Test – MIDAS, pain characteristics, type, duration, frequency, periodicity, headache relief with analgesia, and associated symptoms). QoL was measured using a validated Portuguese version of the SF-36 (Ware and Sherbourne, 1992). The SF-36 was self-administered and contained 36 questions that assessed eight domains (Physical Functioning, Role-Physical, Bodily Pain, General Health, Vitality, Social Functioning, Role-Emotional, and Mental Health). A score at or below the median for each domain of SF-36 was classified as a poor QoL in each domain of this scale. The MIDAS questionnaire (Fragoso, 2002) was used to measure disability caused by headache (minimal, mild, moderate, or severe disability). The PSQI was used to evaluate sleep quality. Those with a score of 11 points or more were considered to have a sleep disorder (Araujo et al., 2015). The BAI was used to measure the degree of anxiety (minimal, mild, moderate, or severe anxiety) (Quintão et al., 2013).

**Figure 1:**
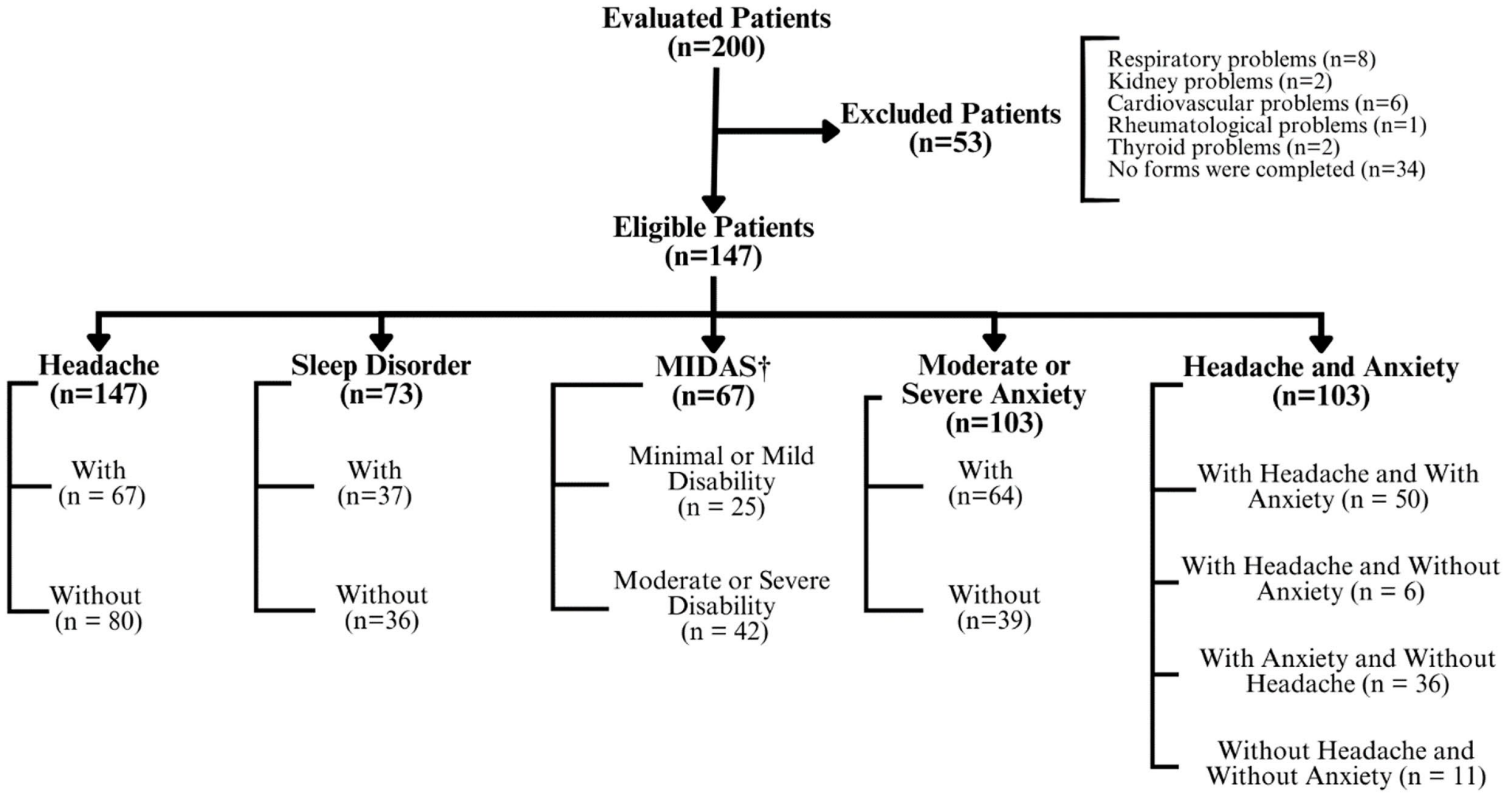
Flowchart of patients in this study. †Migraine Disability Assessment Test (MIDAS).

From August 2020 to March 2023, 200 patients underwent clinical evaluation and completed the SF-36 questionnaire and 53 (26.5%) were excluded because the exclusion criteria. Among the 147 patients included, 135 were diagnosed with confirmed symptomatic long Covid, and 15 with possible symptomatic long Covid and had QoL measured for the eight domains of the SF-36. To evaluate the relationship between QoL and headache, insomnia and anxiety, patients were divided into different groups according to: (1) occurrence of only one condition (patients with headache versus without headache/with versus without sleep disorders/with versus without moderate or severe anxiety); (2) MIDAS result (patients with minimal or mild disability versus moderate or severe disability); (3) association of symptoms (patients with headache and anxiety versus with headache and without anxiety versus with anxiety and without headache versus without headache and without anxiety). Although all forms were sent via instant messaging application, not all patients filled out all the forms (Figure 1).

### 2.3 Statistical analysis

The data were analyzed using GraphPad Prism™ version 5.0 software (GraphPad Software, Inc., San Diego, CA, USA). Continuous variables with a non-normal distribution were compared using the Mann–Whitney *U* test for comparisons of two groups and, the Kruskal–Wallis test for comparisons or three or more groups with *post hoc* Dunn’s test and reported de median and first and third quartiles. Spearman’s rho was used for correlation analysis of ordinal data. We used logistic regression to identify predictors of a poor QoL and calculated the crude and adjusted odds ratios for each exposure factor with 95% confidence intervals (CIs). For multiple logistic regression, univariate analysis was first performed with each of the eight domains of the SF-36 and clinical variables and patient scores, namely: age, scholarity, gender, BAI, PSQI, Visual Analog Pain Scale (VAS), hospitalization due to COVID-19, characteristics of the headache (location (frontal, occipital, holocranial, bitemporal, unilateral), frequency, periodicity, duration, type of pain (pressure, ‘stabbing’, burning and pulsating), presence of associated symptoms, any improvement in pain with medication use, and headache prior to COVID-19). Only independent variables that presented a *p*-value lower than 0.2 in univariate analysis were selected for multivariate analysis. Patients with QOL equal to or lower than the median of each domain of the SF-36 were considered to have a worse QoL outcome in regression analysis. An α level of 0.05 was adopted to reject the null hypothesis.

## 3 Results

In this study long COVID was observed most frequently in women than in men, and the median age was 44.5 years. The majority of participants had not been hospitalized, had a sleep quality disorder, moderate or severe disability attributed by the headache, and moderate or severe anxiety. The median duration of long COVID was 326 days and the most frequently reported symptoms were headache, tiredness, and cognitive complaints (Table 1).

**Table 1:** Characteristics of patients with long COVID in this study.

**Variables**	***N* (%)**
**Gender feminine**	107 (72.79%)
**Average age** (years, SD)	44.5 (±12.18)
**Monthly income**	
<250 dollars	72 (50.35%)
≥250 dollars	71 (49.65%)
**Hospitalization**	
Yes	24 (16.90%)
No	118 (83.10%)
**Average long COVID duration** (days, SD)	326.27 (±241.52)
**Long COVID symptoms**	
Tiredness	64 (43.54%)
Fatigue	38 (25.85%)
Dyspnoea	30 (20.41%)
Myalgia	33 (22.45%)
Chest pain	20 (13.61%)
Back pain	27 (18.37%)
Alopecia	41 (27.89%)
Headache	67 (45.58%)
Insomnia	37 (25.17%)
Anxious symptoms	39 (26.53%)
Anosmia	82 (55.78%)
Ageusia	57 (38.78%)
Paresthesia	27 (18.37%)
Cognitive complaints	59 (40.14%)
**Migraine disability assessment test (MIDAS)**	
Mild or minimum disability	25 (37.31%)
Moderate or severe disability	42 (62.69%)
**Presence of sleep quality disorder**	37 (50.68%)
**Presence of moderate or severe anxiety disorder**	64 (62.14%)

Source: authors themselves.

**Figure 2:**
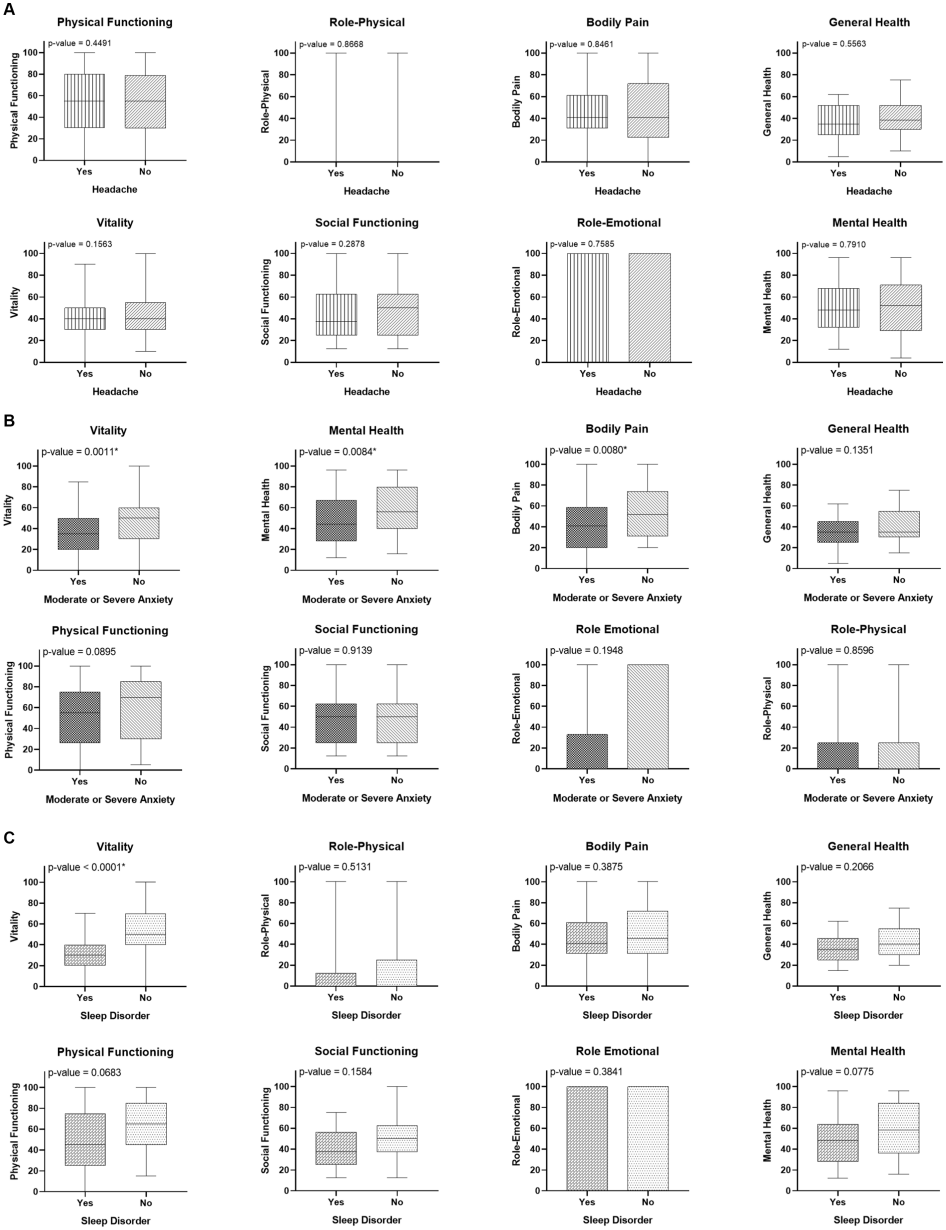
Comparison of the quality of life (QoL) in patients with long COVID **(A)** according to the presence of headache; **(B)** according to the severity of anxiety; and **(C)** according to the presence of sleep disorder.

Patients with sleep disorders had a lower median Vitality score than those without sleep disorders. Patients with moderate or severe anxiety had lower median Bodily Pain, Vitality, and Mental Health scores than patients without moderate or severe anxiety. The domain scores did not differ significantly in patients with or without headache (Figure 2).

**Figure 3:**
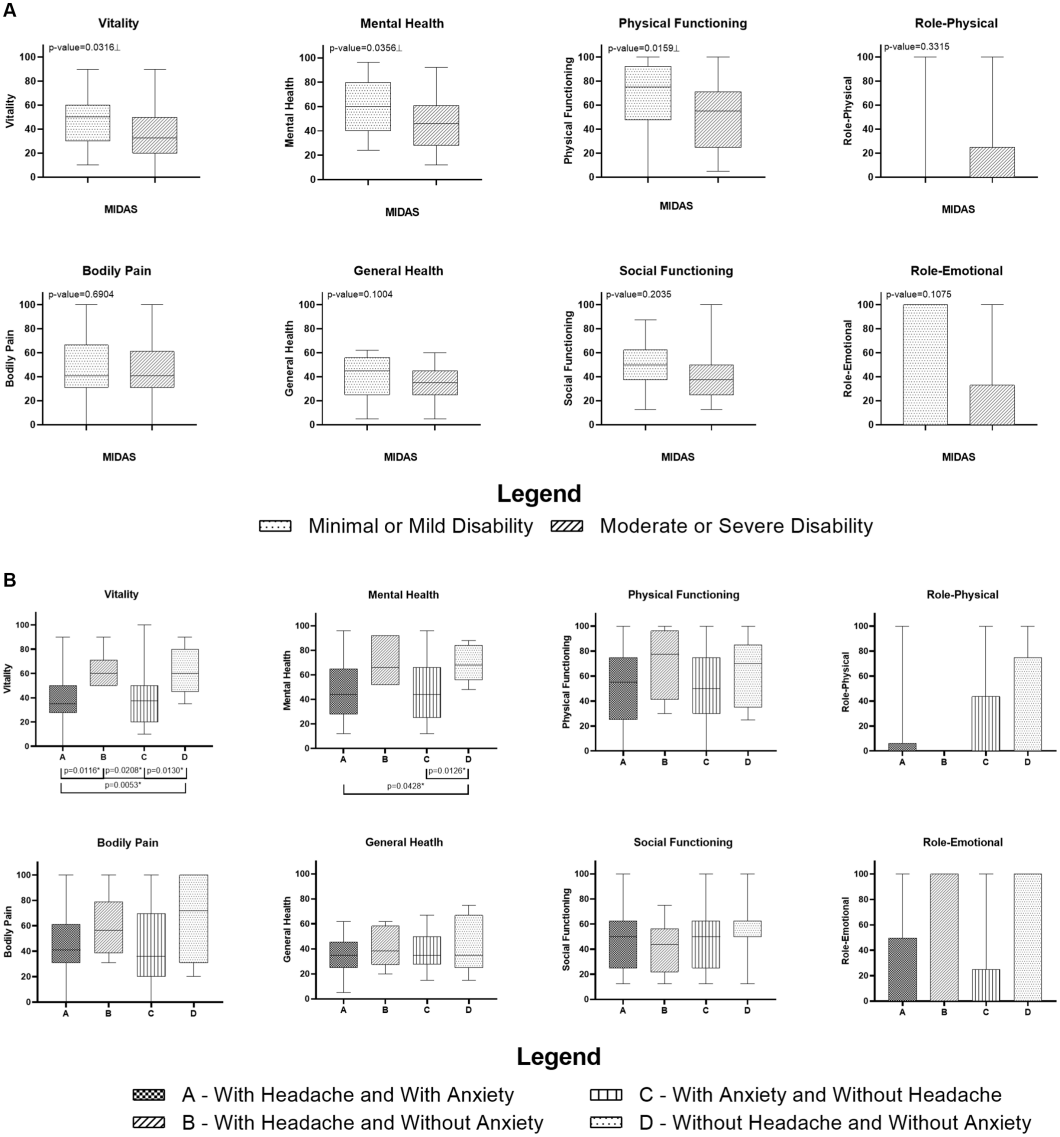
**(A)** Comparison of the Migraine Disability Assessment Test (MIDAS) score with patients headache and long COVID categorized by the domains of 36-Short Form Health Survey; **(B)** Comparison between median and 1st and 3rd quartile of SF-36 domains grouped by the combination between presence or absence of Headache and Anxiety of patients with long COVID. ⊥ Mann–Whitney Test (*p* < 0.05). * Kruskal–Wallis Test with *post hoc* Dunn’s test (*p* < 0.05).

Compared with patients without minimal or mild disability on MIDAS, those with moderate or severe disability had lower median Physical Functioning, Vitality, and Mental Health domain scores (Figure 3).

Examining the interaction between the presence or absence of headache and anxiety, revealed the following: In the Vitality domain, patients with a combination of headache and anxiety had a lower median score than those with experiencing headache alone, and those without headache or anxiety either condition. Additionally, patients with headache alone had a lower median Vitality score than those with anxiety alone, whereas patients without headache or anxiety had a higher median Vitality score than those with anxiety alone.

In the Mental Health domain, patients with both headache and anxiety had a lower median score than those without either condition. Moreover, patients without headache or anxiety had a higher median score than those with anxiety alone (Figure 3).

**Figure 4:**
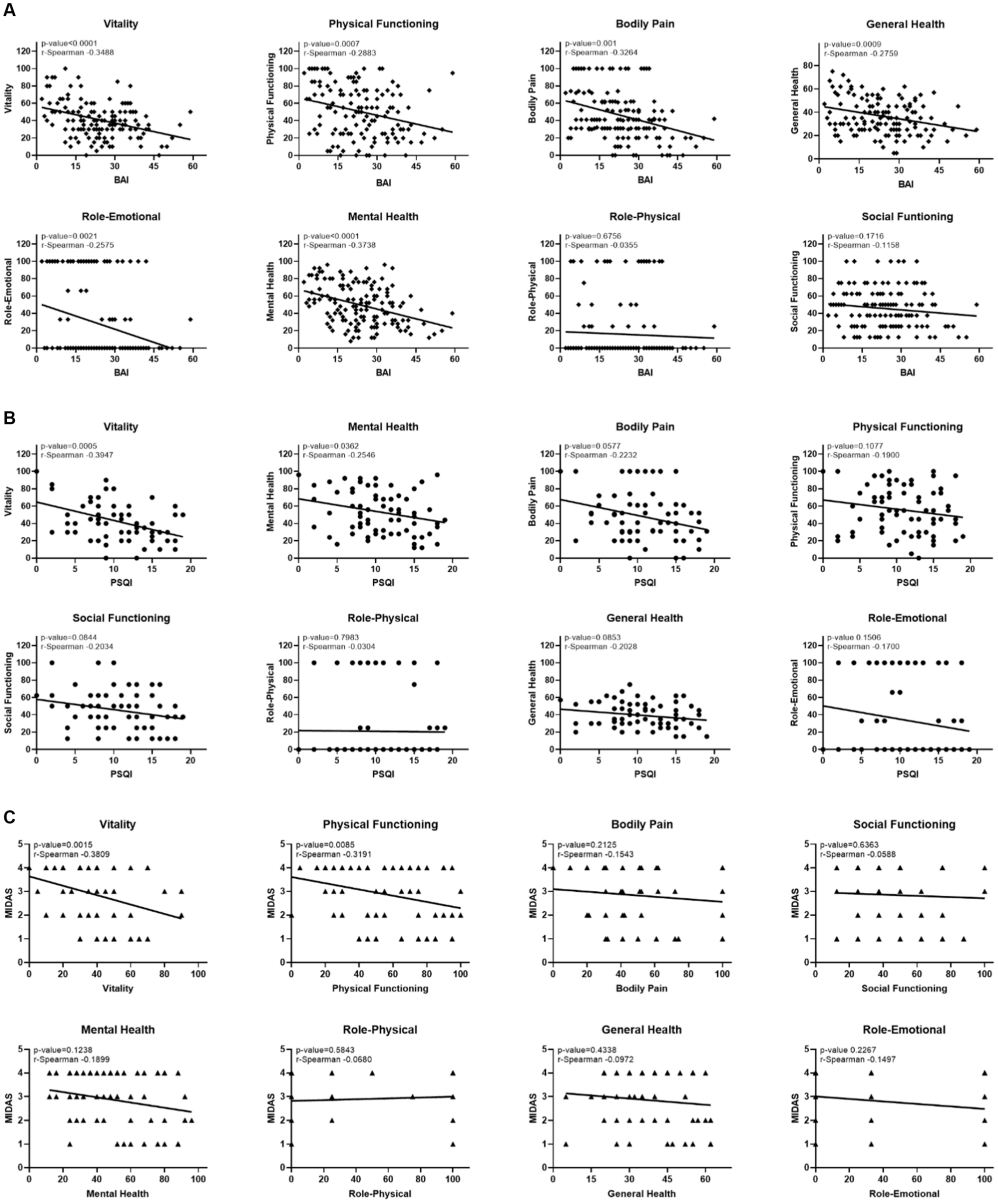
**(A)** Correlation analysis between the domains of the 36-Short Form Health Survey (SF-36) of patients with long COVID and Beck Anxiety Inventory (BAI); **(B)** Correlation analysis between the domains of the SF-36 of patients with long COVID and Pittsburgh Sleep Quality Index (PSQI); **(C)** Correlation analysis between the domains of the SF-36 of patients with long COVID-19 and Migraine Disability Assessment Test (MIDAS).

Correlation analysis revealed lower SF-36 scores in patients with concurrent anxiety, poor sleep quality, and headache. Among patients with long COVID, a direct correlation was observed between anxiety severity and low SF-36 scores within specific domains, namely, Vitality (*p* < 0.0001, Spearman’s rho [ρ] −0.3488), Physical Functioning (*p* = 0.0007, Spearman’s ρ −0.2883), Bodily Pain (*p* = 0.0010, Spearman’s ρ −0.3264), and General Health (*p* = 0.0009, Spearman’s ρ −0.2759). The findings were similar in patients with poor sleep quality. A decline in sleep quality was directly correlated with a lower SF-36 score, notably in the domains of Vitality (*p* = 0.0005, Spearman’s ρ −0.3947) and Mental Health (*p* = 0.0362, Spearman’s ρ −0.3947). Furthermore, headache disability was correlated with lower SF-36 scores in the domains of Vitality (*p* = 0.0015, Spearman’s ρ −0.3809) and Physical Functioning (*p* = 0.0085, Spearman’s ρ −0.3191) (Figure 4).

The analysis of predictors of worse QoL revealed that the presence of a sleep disorder was associated with a four times greater risk of worse Vitality (score less than or equal to the median) (OR 4.47; 95% CI 1.01–19.69; *p* = 0.048) (Table 2). No other risk factors associated with worse outcomes were identified for the other domains of the SF-36 (Supplementary Table S1).

**Table 2:** Multivariable logistic regression of factors associated with poorer quality of life (QoL) in patients with long COVID according to SF-36 domain.

**Outcomes and predictive factors**	**Univariate analysis**				**Multivariate analysis**			
	**OR** **Gross**	**IC 95%**		***p*-value**	**OR** **Adjusted**	**IC 95%**		***p*-value**
		**Lower**	**Upper**			**Lower**	**Upper**	
**Vitality poor (cutoff median ≤ 40)**								
Sleep quality disorder	5.6875	1.6061	20.1408	0.0127	4.4681	1.0140	19.6888	0.0479
Anxiety disorder	4.0154	1.1231	14.3559	0.0580	2.5248	0.5071	12.5699	0.2581
Headache Duration ≥6 h	2.5000	0.8659	7.2181	0.1466	1.3470	0.3102	5.8493	0.6909
Headache location: Holocranial	0.4256	0.1506	1.2027	0.1724	0.9010	0.1805	4.4967	0.8989

Source: authors themselves.

## 4 Discussion

In our study, we observed that patients with long COVID have experienced a significant effect on their QoL, specifically in the Vitality and Mental Health domains. It is worth noting that the presence of sleep disorders, anxiety, or debilitating headaches, either individually or in combination, has been associated with even worse Vitality outcomes in these patients. Additionally, our observations imply that an inadequate sleep quality pattern, as assessed using the PSQI, could potentially function as a risk factor, demonstrating a fourfold likelihood of worsening Vitality in these individuals. The Mental Health domain involves susceptibility to anxiety and incapacitating headaches, whether experienced concurrently or in isolation. Correlation analysis revealed a potential association between a decline in sleep quality and the corresponding deterioration in Mental Health. Specifically, patients with long COVID and anxiety reported lower QoL in the Vitality, Mental Health, and Bodily Pain domains, whereas those with debilitating headaches reported worse Physical Functioning, Vitality, and Mental Health. Through our correlation analysis examining long COVID individually associated with headache, anxiety, and insomnia, we discovered a consistent trend of decreased QoL across all domains.

The Vitality domain of SF-36 delineates the appraisal of an individual’s state of well-being by scrutinizing four distinct parameters within this metric. Individuals with lower scores in this domain commonly encounter symptoms such as fatigue, lethargy, indisposition, and diminished efficacy in the execution of routine activities of daily living. Among the array of symptoms exhibited by individuals experiencing the effects of long COVID, fatigue and tiredness have emerged prominently as primary manifestations (Al-Ramadan et al., 2021). In an exhaustive meta-analysis spanning 74 studies, findings indicated that fatigue was reported as a prevalent symptom of long COVID in 32% of study participants. Although women reported fatigue more frequently, no statistically significant differences were observed between the reporting patterns of men and women. Additionally, older adults consistently reported a higher prevalence of fatigue (Ceban et al., 2022).

Several viral illnesses have historically been associated with chronic fatigue. Individuals affected by the initial SARS epidemic in 2003 documented persistent and chronic fatigue, disruptions in sleep patterns, and manifestations of depression (Sukocheva et al., 2022). Notably, patients in Canada reported enduring challenges in occupational functioning 1 to 3 years post-infection, primarily attributed to the presence of sustained and chronic fatigue (Moldofsky and Patcai, 2011).

Long COVID and chronic fatigue syndrome/myalgic encephalomyelitis (CFS/ME), both disease induced by viral agent, commonly exhibit cognitive, psychological, and sleep-related alterations, as well as chronic fatigue, among other shared characteristics (Qanneta, 2022). CFS/ME is a condition with multifaceted origins, challenging diagnostic procedures, and intricate multidisciplinary management. Both conditions share pathophysiological parallels, including modifications in oxidative stress, autoimmune pathways, and mitochondrial dysfunction (Paul et al., 2021). Owing to these congruities, apprehensions arise regarding the potential for long-term repercussions of long COVID, mirroring those of CFS/ME, potentially culminating in a diminished QoL.

The effect of insomnia on QoL is well described in the literature. A prospective Italian study of 525 individuals with long COVID reported that sleep disorders evidenced by the PSQI could predict worse SF-36 scores (Mastrorosa et al., 2023). In another study, poor sleep quality, daytime sleepiness, sleep inertia, naps, insomnia, sleep apnoea, and nightmares were observed in patients with long COVID compared with those in the acute phase (Scarpelli et al., 2023). Although no study has reported an association between sleep disorders and Vitality, we believe that patients with sleep disorders may experience daytime sleepiness, indisposition, and slowness, all of which can affect Vitality. It is likely that other diverse clinical conditions present in long COVID periods, such as dyspnoea and fatigue, may affect sleep quality.

Mental Health was the second most affected in patients with long COVID. Anxiety is one of the main factors affecting QoL (Román-Montes et al., 2023). Patients of this study who have anxiety showed worse Vitality, Mental Health, and Bodily Pain. Similar results have been reported previously (Pérez Catalán et al., 2023). A meta-analysis that included 4,828 patients with long COVID in 12 studies showed that 38% of the sample had poor QoL attributed to anxiety/depression (Malik et al., 2022). These patients may experience cognitive dysfunction, reduced usual activities, and self-care highlights, which may affect their ability to participate in social functions (Tabacof et al., 2022). It is reasonable to consider that patients with anxiety may have social phobias and socialization difficulties, which may affect the social functions that they perform in society as well as their mental and physical health.

A recent cross-sectional study published by our group of 288 patients with long COVID showed that the presence of anxiety is a risk factor for poor sleep quality, and that patients with sleep disorders more frequently report symptoms of anxiety (Paranhos et al., 2023). Anxiety and insomnia have common pathophysiological mechanisms. Dysregulation of neurotransmitters such as cholinergic and gamma-aminobutyric acid (GABA) can cause hyperexcitation, which associated with insufficient sleep can disrupt the functioning of the corticolimbic circuit and impair the regulation of emotions, including anxiety (Riemann et al., 2010; Blake et al., 2018). It is reasonable to conclude that the association between these two conditions may partly explain the poor QoL of the patients in this study.

The disabling and severe headache were conditions associate to worse QoL of our patients. Similar results were found in a study from Indonesia with 215 participants, which showed that some factors can predict worse QoL in patients with long COVID and headache, such as: recent SARS-CoV-2 infection in less time (3–12 months), earlier onset of headache (1–4 weeks after infection), patients who had taken pain medication, longer duration of headache, daily frequency, combination of throbbing, grinding, and stabbing headache, severe headache, and associated symptoms (Mutiawati et al., 2022). Another study reported that a previous headache history can worsen the QoL of patients with long COVID when compared to any previous migraine (Thawani et al., 2022). Although these studies did not report headache characteristics and predictive factors for worse QoL in the SF-36 domains, our results point in the same direction. Additionally, a study published by our research group reported that patients with long COVID who presented with disabling headaches with severe characteristics tended to have chronic pain (Rodrigues et al., 2023). A pronounced headache is likely to constrain individuals’ day-to-day pursuits, encompassing professional obligations, academic endeavors, domestic responsibilities, and other facets of daily life. This has the potential to exacerbate their overall QoL.

The pathophysiology of headaches in patients with long COVID is poorly understood. It is likely that the direct brain invasion of the virus, cytokines, interleukin storms, persistent activation of the immune system, and pro-inflammatory state in long COVID may be associated with persistent and disabling headaches (Bolay et al., 2021; Dani et al., 2021; Karadaş et al., 2021; Martelletti, 2021; Trigo et al., 2021).

Due to these enduring and protracted changes, long COVID has emerged as a global public health challenge. Following viral contraction, numerous patients may experience diminished QoL and encounter impediments in executing fundamental activities, such as employment, household responsibilities, and self-care (Líška et al., 2022; Moens et al., 2022). This erosion of autonomy and functional capacity may act as a potential catalyst for heightened anxiety, insomnia, headaches, and other concurrent health issues, thereby exacerbating poor QoL and setting a detrimental cycle. The prevailing sentiments of profound and persistent fatigue, coupled with non-restorative sleep and psychological disturbances, are potential factors that markedly impede the motivation of individuals to actively engage in their previous social roles. These concurrent factors can invariably lead to a compromised sense of autonomy, diminished ability to generate income, and limited capacity to fulfill familial and communal obligations. These changes can substantially worsen QoL.

Concerted efforts should be directed toward dismantling this deleterious cycle and reinstating the QoL of individuals with long COVID through rehabilitative interventions (WHO, 2023). In the Amazon context, several peculiarities must be considered when attempting to mitigate the long-term effects of the SARS-CoV-2 infection. The increase in deforestation, fires, and climate change in the Amazon region alters the human–forest relationship. Every day, emerging Amazon pathogens with the potential to contaminate water, air, soil, and food are discovered. In addition to these factors, precarious housing conditions, lack of basic sanitation, among others, make this population even more vulnerable to contracting these new diseases (Santos et al., 2010; Garnelo, 2019; Alves de Oliveira et al., 2021; Codeço et al., 2021; Feng et al., 2021). Currently, little is known about the association between long COVID and other infectious diseases emerging from the Amazon, such as dengue, Zika virus, chikungunya, yellow fever, and Chagas disease. It is likely that patients with these diseases, previously or concomitantly with a long COVID have peculiar characteristics and require specific care in rehabilitation therapies.

Among the limitations of this study, it is important to highlight that the research was conducted with a small sample and that the sampling was for convenience and from a single center, which may not represent the QoL of patients from other locations. Not all participants completed all the standardized protocols sent via instant messaging application due to, among other factors, communication problems. There are some validated questionnaires to assess QoL, and although the SF-36 is a widely used and validated questionnaire, there is still no consensus on which questionnaire to use to assess the QoL of patients with long COVID. Most studies reported the results using summary measures of the SF-36 (Physical and Mental Components); in this study, the results were reported using the SF-36 domains. This may impair the comparison of the results obtained in this study with those of other studies in the current literature.

## 5 Conclusion

In conclusion, QoL was affected by long COVID-19 and the Vitality domain was the most affected by the presence of headache, anxiety, and sleep disorder. The presence of a sleep disorder was associated with a fourfold increase in the risk of low Vitality. Through our correlation analysis examining long COVID individually associated with headache, anxiety, and insomnia, we discovered a consistent trend of decreased quality of life across domains. It is likely that a combination of these conditions reduces QoL, preventing patients from performing activities of daily living. This study is the first to describe the QoL of patients with an association between long COVID and headache, sleep disorders and anxiety in the Amazon, highlighting the need for prospective studies to assess the QoL of these patients at different times.

## Data availability statement

The raw data supporting the conclusions of this article will be made available by the authors, without undue reservation.

## Ethics statement

The studies involving humans were approved by Ethics Committee for Human Subjects Research of the State University of Pará (approval number 3,619,141). The studies were conducted in accordance with the local legislation and institutional requirements. The participants provided their written informed consent to participate in this study.

## Author contributions

AR: Conceptualization, Data curation, Formal analysis, Funding acquisition, Investigation, Methodology, Project administration, Resources, Software, Supervision, Validation, Visualization, Writing – original draft, Writing – review & editing. AP: Writing – original draft, Writing – review & editing. LS: Writing – original draft, Writing – review & editing. SX: Writing – original draft, Writing – review & editing. CS: Writing – original draft, Writing – review & editing. RS: Writing – original draft, Writing – review & editing. LV: Writing – original draft, Writing – review & editing. IP: Writing – original draft, Writing – review & editing. TP: Writing – original draft, Writing – review & editing. PT: Writing – original draft, Writing – review & editing. CR: Writing – original draft, Writing – review & editing. BL: Writing – original draft, Writing – review & editing. VP: Writing – original draft, Writing – review & editing. PV: Writing – review & editing, Writing – original draft. JQ: Writing – original draft, Writing – review & editing, Conceptualization, Data curation, Formal analysis, Funding acquisition, Investigation, Methodology, Project administration, Resources, Software, Supervision, Validation, Visualization. LF: Writing – original draft, Writing – review & editing, Conceptualization, Data curation, Formal analysis, Funding acquisition, Investigation, Methodology, Project administration, Resources, Software, Supervision, Validation, Visualization.
