# Supplementary material for: Effect of long COVID-19 syndrome on health-related quality of life: a cross-sectional study
Source: Front Psychol. 2024 May 30;15:1394068. doi: 10.3389/fpsyg.2024.1394068 (PMC11169885; doi:10.3389/fpsyg.2024.1394068)
Supplement: Supplementary file 1 [file Table_1.docx]

**Table Supplementary 1:** Multivariable logistic regression of factors associated with poorer quality of life (QoL) in patients with long COVID according to SF-36 domain.

| **OUTCOMES AND PREDICTIVE FACTORS** | **Univariaty Analysis** | | | | **Multivariate Analysis** | | | |  |
| --- | --- | --- | --- | --- | --- | --- | --- | --- | --- |
|  | **OR_Gross_** | **IC 95%** | | **p-value** | **OR_Adjusted_** | **IC 95%** | | **p-value** |  |
|  |  | **Lower** | **Upper** |  |  | **Lower** | **Upper** |  |  |
| **Role-Physical (cutoff median ≤ 0)** | | | | | | | | | |
| Headache Frequency ≥2 twice a week (Yes/No) | 0.1282 | 0.0154 | 1.0641 | 0.0459 | 0.0876 | 0.0089 | 0.8586 | 0.0365 |  |
| Headache Location: Frontal (Yes/No) | 2.9545 | 0.8124 | 10.7450 | 0.1668 | 3.3403 | 0.7752 | 14.3929 | 0.1056 |  |
| Headache Location: Unilateral (Yes/No) | 0.2230 | 0.0636 | 0.7817 | 0.0344 | 0.1676 | 0.0401 | 0.7006 | 0.0144 |  |
| **Mental Health (cut off median ≤ 48)** | | | | | | | | | |
| Anxiety Disorder (Yes/No) | 3.6765 | 0.9889 | 13.6678 | 0.0894 | 3.8802 | 0.8431 | 17.8573 | 0.0817 |  |
| Headache prior to COVID-19 (Yes/No) | 0.3889 | 0.1386 | 1.0910 | 0.1195 | 0.2773 | 0.0788 | 0.9758 | 0.0457 |  |
| Headache Location: Holocranial (Yes/No) | 0.3333 | 0.1169 | 0.9503 | 0.0677 | 0.3345 | 0.0914 | 1.2238 | 0.0980 |  |
| Localização da Cefaleia: Occipital (Yes/No) | 0.4000 | 0.1439 | 1.1122 | 0.1291 | 0.5052 | 0.1419 | 1.7982 | 0.2918 |  |
| **General Health (cut off median ≤ 35)** | | | | | | | | | |
| Headache Location: Occipital (Yes/No) | 2.1923 | 0.7941 | 6.0524 | 0.2038 | 2.4373 | 0.8372 | 7.0950 | 0.1022 |  |
| Headache Periodicity ≥2 shifts of the day (Yes/No) | 0.3200 | 0.1062 | 0.9644 | 0.0730 | 0.2934 | 0.0938 | 0.9183 | 0.0352 |  |
| **Social Functioning (cut off median ≤ 37.5)** | | | | | | | | | |
| Headache Periodicity ≥2 shifts of the day (Yes/No) | 0.1803 | 0.0549 | 0.5917 | 0.0072 | 0.1775 | 0.0528 | 0.5966 | 0.0052 |  |
| Headache Location: Occipital (Yes/No) | 2.1923 | 0.7941 | 6.0524 | 0.2000 | 2.48 | 0.7520 | 6.7607 | 0.1467 |  |
| **Role-Emotional (cutoff median ≤ 0)** | | | | | | | | | |
| Some improvement of Headache with medication (Yes/No) | 0.1748 | 0.0355 | 0.6817 | 0.0441 | 0.5143 | 0.0401 | 6.6017 | 0.6096 |  |
| Improvement of Headache without medication (Yes/No) | 9.3704 | 1.1232 | 78.1716 | 0.0379 | 0.5491 | 0.1801 | 1.6739 | 0.2918 |  |
| Headache prior to COVID-19 (Yes/No) | 0.4167 | 0.1465 | 1.1853 | 0.1632 | 3.7526 | 0.1434 | 98.2161 | 0.4272 |  |
| Headache Periodicity ≥2 shifts of the day (Yes/No) | 0.4221 | 0.1434 | 1.2427 | 0.1914 | 0.5101 | 0.1641 | 1.5860 | 0.2448 |  |
| **Bodily-Pain (cut off median ≤ 41)** | | | | | | | | | |
| Headache Location: Holocranial (Yes/No) | 0.4174 | 0.1488 | 1.1706 | 0.1566 | 0.3679 | 0.1202 | 1.1264 | 0.0799 |  |
| Improvement of Headache without medication (Yes/No) | 5.0000 | 0.9932 | 25.1711 | 0.0771 | 4.4993 | 0.8297 | 24.4001 | 0.0813 |  |
| Headache Periodicity ≥2 shifts of the day (Yes/No) | 0.3191 | 0.1071 | 0.9509 | 0.0694 | 0.3393 | 0.1063 | 1.0832 | 0.0680 |  |
| **Physical Functioning (cutoff median ≤ 55)** | | | | | | | | | |
| Schooling ≥9 years of schooling (Yes/No) | 0.1813 | 0.0200 | 1.6441 | 0.2129 | 0.1292 | 0.0127 | 1.3131 | 0.0837 |  |
| Headache Location: Frontal (Yes/No) | 0.4000 | 0.1439 | 1.1122 | 0.1291 | 0.3307 | 0.1078 | 1.0147 | 0.0531 |  |
| Improvement of Headache without medication (Yes/No) | 3.5217 | 0.8512 | 14.5713 | 0.1379 | 2.6036 | 0.5877 | 11.5336 | 0.2076 |  |

Source: authors themselves.
